# Responsive neurostimulation for focal motor status epilepticus

**DOI:** 10.1002/acn3.51318

**Published:** 2021-05-06

**Authors:** Jimmy C. Yang, Nitish M. Harid, Fábio A. Nascimento, Vasileios Kokkinos, Abigail Shaughnessy, Alice D. Lam, M. Brandon Westover, Thabele M. Leslie‐Mazwi, Leigh R. Hochberg, Eric S. Rosenthal, Andrew J. Cole, Robert M. Richardson, Sydney S. Cash

**Affiliations:** ^1^ Department of Neurosurgery Massachusetts General Hospital Harvard Medical School Boston Massachusetts USA; ^2^ Department of Neurology Massachusetts General Hospital Harvard Medical School Boston Massachusetts USA; ^3^ Center for Neurotechnology and Neurorecovery Massachusetts General Hospital Boston Massachusetts USA

## Abstract

No clear evidence‐based treatment paradigm currently exists for refractory and super‐refractory status epilepticus, which can result in significant mortality and morbidity. While patients are typically treated with antiepileptic drugs and anesthetics, neurosurgical neuromodulation techniques can also be considered. We present a novel case in which responsive neurostimulation was used to effectively treat a patient who had developed super‐refractory status epilepticus, later consistent with epilepsia partialis continua, that was refractory to antiepileptic drugs, immunomodulatory therapies, and transcranial magnetic stimulation. This case demonstrates how regional therapy provided by responsive neurostimulation can be effective in treating super‐refractory status epilepticus through neuromodulation of seizure networks.

## Introduction

Status epilepticus can result in significant mortality or debilitating cognitive and motor sequelae in survivors. Prognosis for patients affected by status epilepticus (SE) is crucially dependent on reducing the duration of seizure activity.^1^ When uncontrolled by first‐ and second‐line antiepileptic drugs (AEDs), SE is deemed refractory (RSE) and progresses to super‐refractory (SRSE) when it persists after 24 h of general anesthesia.^2^ Although acute brain injury can result in SRSE, it can also occur in patients without a history of injury or epilepsy, termed new‐onset refractory status epilepticus (NORSE).^2^, ^3^ Notably, no clear best treatment paradigm exists for RSE.^4^

SE can be further defined and classified based on electroclinical characteristics, and much of the current data tying seizure activity to long‐term consequences are related to convulsive SE.^5^ Epilepsia partialis continua (EPC) is a type of focal motor SE that can arise from multiple etiologies, which can affect its prognosis.^6^, ^7^, ^8^ In some cases, EPC can be tied to prior diagnoses of epilepsy or SE, and its duration has been linked as an independent predictor of mortality.^9^, ^10^, ^11^

Neurosurgery has been considered an option for the treatment of RSE and SRSE, and both resection and neuromodulation have been used.^12^, ^13^, ^14^ Surgery is typically considered after medical therapies have been exhausted, which may result in less optimal outcomes.^15^ Neuromodulation can be additionally implemented in cases where lesions are diffuse or involve eloquent regions.

Here we present the case of a patient with NORSE that later was consistent with EPC, which was refractory to immunomodulatory therapy and repetitive transcranial magnetic stimulation (rTMS). No clear lesion was identified on imaging, and responsive neurostimulation (RNS), implanted after intracranial electrocorticography and stereoelectroencephalography (stereo‐EEG), was used to treat her SE. We demonstrate the utility of RNS as a tool in challenging cases of nonlesional SRSE or EPC.

## Case

The patient is a 22‐year‐old woman with a history of vesicoureteral reflux and related nephropathy, who initially presented with left visual field distortions and severe headache, which developed into left arm myoclonic jerking, left eye deviation, and left head version with intermittent impairment of awareness, associated with electrographic seizures in the right posterior region on scalp EEG.

**Figure 1:**
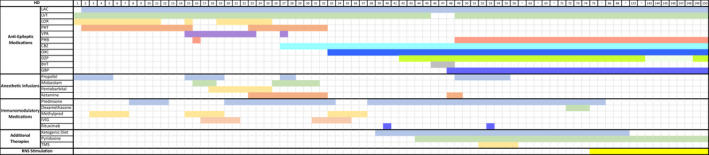
Color‐coded tracking of antiepileptic medication administration, anesthetic infusions, immunomodulatory medications, and additional therapies used in the patient's treatment. RNS stimulation was started on HD75. LAC, lacosamide; LVT, levetiracetam; LOR, lorazepam; PHT, phenytoin (or fosphenytoin); VPA, valproate; PHB, phenobarbital; CBZ, clobazam; OXC, oxcarbazepine; DZP, diazepam; BVT, brivaracetam; GBP, gabapentin; Methylpred, methylprednisolone; TMS, transcranial magnetic stimulation; HD, hospital day.

She was treated with levetiracetam and lorazepam but continued to have electrographic and clinical seizures (Fig. 1). Additional AEDs were added without success; thus, she was sedated with propofol to achieve burst suppression that required intubation. Initial imaging studies did not demonstrate a clear lesion, and cerebrospinal fluid and serum autoimmune, paraneoplastic, metabolic, neoplastic, toxicologic, and infectious studies were unremarkable (Table 1). Eventually, her exam improved to the point where she had a normal neurologic exam. However, on hospital day 15, her seizures recurred, requiring multiple sedatives (Fig. 1). Due to concern for an autoimmune etiology, she was empirically treated with methylprednisolone and IVIG, without significant improvement.

**Table 1:** Results of Laboratory Tests.

HD	Study	Sample origin	Result	Notes
Autoimmune/paraneoplastic studies				
2	Mayo encephalopathy/autoimmune evaluation	Serum	Negative	
2	Mayo encephalopathy/autoimmune evaluation	CSF	Negative	Additionally negative on HD18, 31
2	ANA	Serum	Negative	Additionally negative on HD16
2	ANCA	Serum	Negative	
2	Oligoclonal banding	Serum	Negative	Additionally negative on HD18
2	Oligoclonal banding	CSF	Negative	Additionally negative on HD18, 31
15	Thyroperoxidase antibody	Serum	Negative	
15	Antithyroglobulin	Serum	Negative	
16	Mayo encephalopathy/autoimmune evaluation	Serum	Positive for N‐type calcium channel antibody^1^	
16	SSA antibody	Serum	Negative	
16	SSB antibody	Serum	Negative	
16	Smith antibody	Serum	Negative	
16	RNP antibody	Serum	Negative	
16	Anti‐DNA (double stranded) antibody	Serum	Negative	
18	Mayo Paraneoplastic/Autoantibody Evaluation	CSF	Negative	Additionally negative on HD31
30	Voltage‐Gated Potassium Channel Antibody	Serum	Negative	
30	Mayo Encephalopathy/Autoimmune Evaluation	Serum	Positive for GAD65 Antibody and N‐type Calcium Channel Antibody^1^	
Metabolic studies				
1	TSH	Serum	Within normal limits	
2	Free T4	Serum	Within normal limits	
10	TSH	Serum	Within normal limits	
Neoplastic studies				
2	CSF flow cytometry	CSF	No evidence of immunophenotypic abnormalities	
2	CSF cytology	CSF	Negative for malignant cells	
3	Neuron‐specific enolase	Serum	Negative	
31	Flow cytometry, peripheral blood	Blood	Within normal limits	
Infectious studies				
1	COVID‐19 PCR	Nasopharyngeal	Negative	Additionally negative on HD15, 25
2	Ehrlichia/anaplasma PCR	Serum	Negative	
2	West Nile virus PCR	CSF	Negative	
2	Enterovirus PCR	CSF	Negative	
2	Eastern equine encephalitis IgG, IgM	CSF	Negative	Additionally negative on HD18
2	Epstein–Barr virus, IgM	Serum	Equivocal	
2	Epstein–Barr virus, IgG	Serum	Positive	
2	Varicella Zoster virus PCR	CSF	Negative	Additionally negative on HD18
2	Herpes simplex virus 1/2 PCR	CSF	Negative	Additionally negative on HD18
2	Cryptococcal antigen	CSF	Negative	
2	Lyme IgG	CSF	Negative	
2	VDRL	CSF	Negative	
17	HIV 1/2 antigen and antibody	Serum	Negative	
31	RT‐QuIC for sporadic Creutzfeldt–Jakob disease	CSF	Negative	
Toxicology studies				
1	Toxicology screen	Urine	Positive for benzodiazepines	Had been treated with lorazepam

Table of notable autoimmune, paraneoplastic, metabolic, neoplastic, and infectious studies that were conducted. CSF, cerebrospinal fluid. HD, hospital day.

^1^ Note that positive serum tests for GAD65 and N‐type calcium channel antibody were considered false positives in the setting of prior IVIG administration.

**Figure 2:**
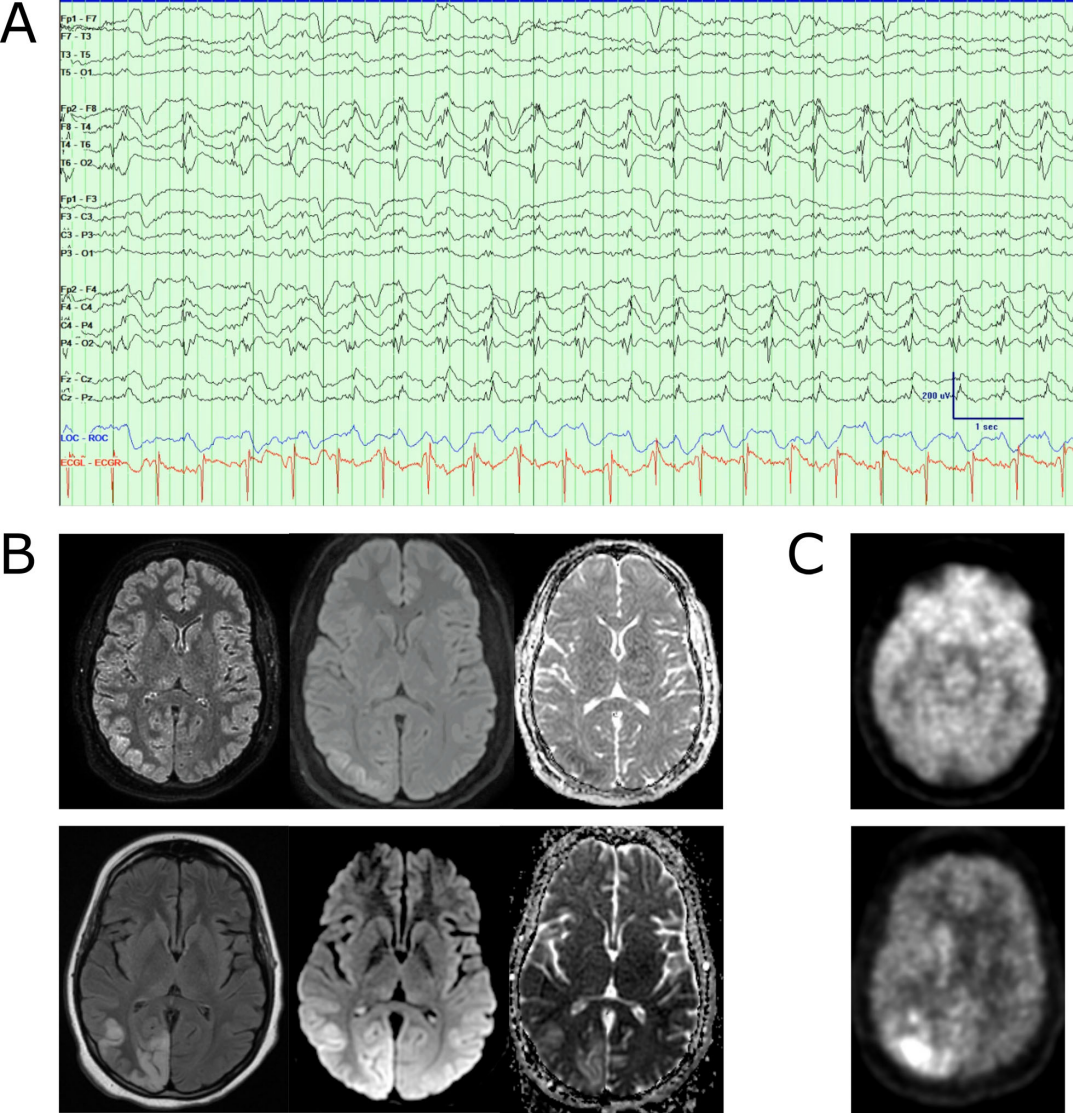
(A) Example of lateralized periodic discharges in the right posterior quadrant in a bipolar montage. Low frequency filter: 1 Hz, High frequency filter: 70 Hz, Notch: 60 Hz, sampling frequency: 512 Hz. Blue tracing demonstrates eye movements, and red tracing is concurrent ECG. (B) Two sets of MRIs, top row obtained on hospital day 1, with FLAIR, DWI, and ADC sequences (left to right). Bottom row obtained on hospital day 58. These demonstrate hyperintensity over the right occipital and parietal lobes. (C) Two PET scans, top image obtained on hospital day 29, bottom image obtained on hospital day 47. While top image demonstrates global hypometabolism in the bilateral parietal and occipital lobes, the bottom image suggests hypermetabolism in the right parietal and occipital lobes.

She was transferred to Massachusetts General Hospital on hospital day 26, while on anesthetic infusions. After anesthetics were discontinued, she could open her eyes to voice but could not regard nor follow commands, and she had flexion movements to stimulation in the upper and lower extremities, which were brisker on the right. EEG showed right occipital periodic discharges (Fig. 2A); additional AEDs were added, and a ketogenic diet was initiated.

Imaging studies were unrevealing. Whole‐body PET did not reveal underlying malignancy. Serial MRIs demonstrated DWI and FLAIR changes thought to be sequelae of seizures (Fig. 2B). Initial brain PET suggested regional hypometabolism of the bilateral occipital and parietal lobes, but repeat PET on hospital day 47 showed hypermetabolism in the right parietal and occipital lobes, presumably due to her ongoing seizure activity (Fig. 2C).

To focally disrupt ongoing seizure activity noninvasively, she received 10 total sessions over 5 days of 1‐Hz rTMS to the right parietal and occipital regions starting on hospital day 52, with each session lasting 30 min and at 80% machine output, due to the motor threshold being indeterminate at maximal machine output. This regimen was adapted based on prior reports.^16^, ^17^ Prior to treatment, she was able to follow axial and appendicular commands, but she continued to demonstrate continuous clonic movements of the left neck, shoulder, proximal arm, and leg, without purposeful movement, which occurred both while awake and asleep. Clinically, this appeared consistent with a focal motor SE or EPC. Given the inadequate response to these therapies, chronic neuromodulation was considered.

With a nonlesional MRI, intracranial EEG was planned to better understand whether a focal or diffuse region was involved. On hospital day 65, she underwent a craniotomy for placement of subdural strips that covered the right parietal and occipital lobes, as well as cortical biopsy of the FLAIR hyperintense region. Pathology demonstrated diffuse reactive gliosis and neuronal loss, felt to be consistent with her prolonged SE.

**Figure 3:**
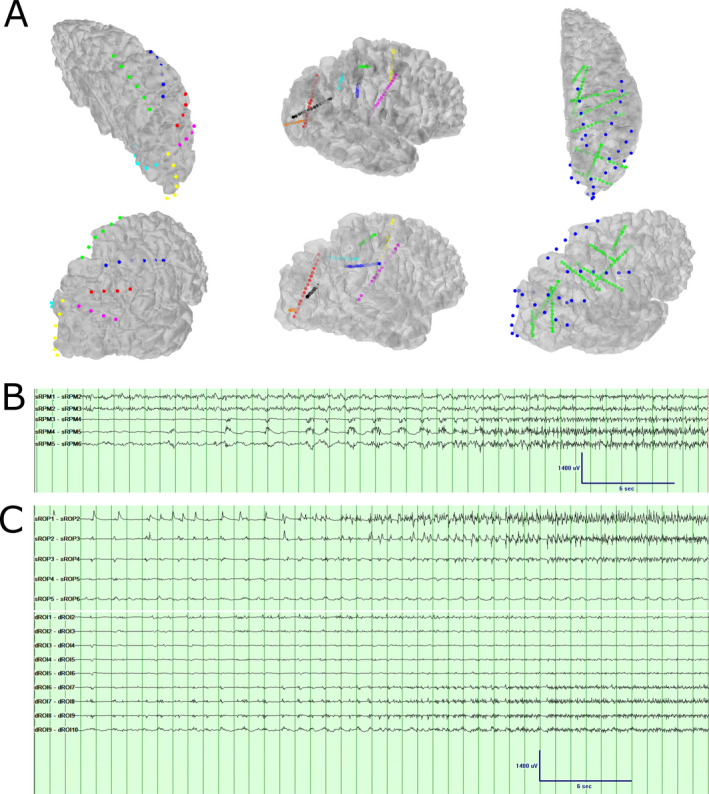
(A) 3D reconstruction of strip and depth electrodes implanted. The first column shows *strip electrodes*: RPM, right parietal mesial (green). RPA, right parietal anterior (blue); RPL, right parietal lateral (red); ROM, right occipital mesial (cyan); ROP, right occipital posterior (yellow); ROL, right occipital lateral (magenta). The second column shows *depth electrodes*: RSMA, right supplementary motor area (yellow); RFS, right frontal superior (green); RFI, right frontal inferior (blue); RPT, right parietal (cyan); ROS, right occipital superior (black); ROI, right occipital inferior (orange); RPO, right parietal‐occipital (red); RCM, right centromedian thalamic nucleus (magenta). The third column shows the relation between strip electrode coverage (blue) and depth electrode coverage (green). (B) One seizure pattern seen with invasive monitoring, primarily involving the right parietal mesial strip (sRPM) on contacts 4–6. Low‐frequency filter: 0.5 Hz, High‐frequency filter: 70 Hz, Notch: 60 Hz, sampling frequency: 512 Hz. (C) Second seizure pattern seen with invasive monitoring, primarily involving the right occipital posterior strip (sROP) on contacts 1–3, as well as the right occipital inferior depth on contacts 7–10. (dROI) Low‐frequency filter: 0.5 Hz, High‐frequency filter: 70 Hz, Notch: 60 Hz, sampling frequency: 512 Hz.

Due to concern that the seizure focus needed to be further localized, she underwent placement of stereo‐EEG electrodes on hospital day 71, which covered the deep structures around the right frontoparietooccipital regions as well as the centromedian (CM) thalamic nucleus (Fig. 3A). Electrographic onsets were in the right occipital posterior region and independently in the right mesial parietal area (Fig. 3B and C). These regions were very active, with seizures from the occipital region occurring every 2–4 min and from the mesial parietal region every 3–5 min. While seizures could be seen independently at both sites, there were some events in which the seizures could progress from the occipital region to the parietal region. Interictally, these same regions demonstrated abundant epileptiform discharges, though occasional to frequent discharges could also be seen in the lateral parietal and frontal regions. There was no EEG correlate to EMG‐recorded motor activity on scalp, strip, or depth electrodes, even with averaging to the motor activity. Ultimately, this finding was hypothesized to be either undersampling of the cortex or the presence of a subcortical source of myoclonic activity. Given her uncontrolled right occipital seizure activity, another hypothesis was that the myoclonic activity represented undetected downstream or propagated activity.

**Figure 4:**
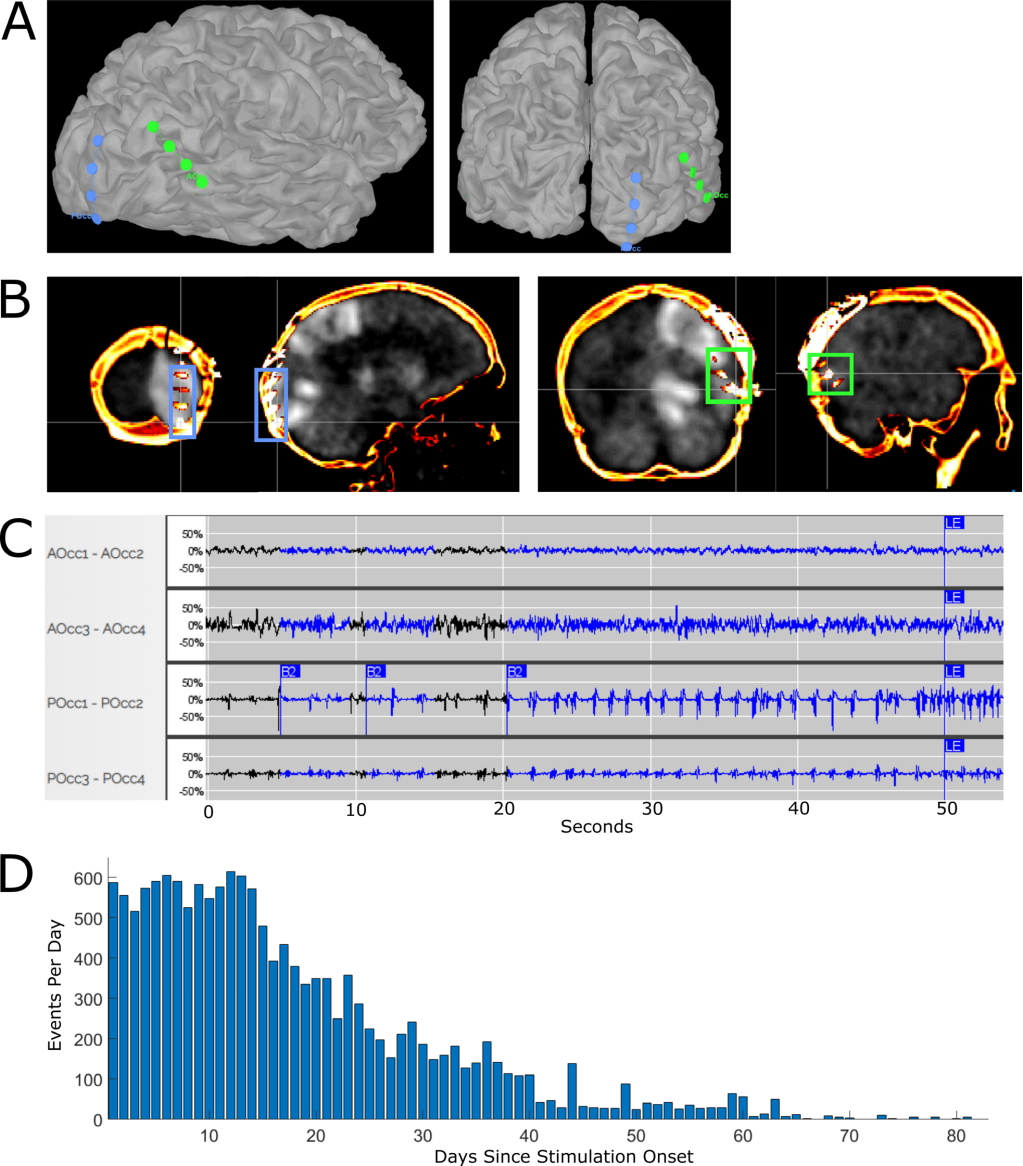
(A) 3D reconstruction of RNS strip electrodes on the cortical surface. Green electrode, AOcc, corresponds to the anterior occipital strip. Blue electrode, POcc, corresponds to the posterior occipital strip. (B) Coronal (left) and sagittal (right) views of RNS strip electrodes co‐registered onto PET brain scan obtained on hospital day 57. Blue box corresponds to the location of the anterior occipital strip, and green box corresponds to the location of the posterior occipital strip. (C) Demonstration of an event captured by the responsive neurostimulation device in bipolar montage, seen primarily on the posterior occipital strip, contacts 1–2. AOcc, anterior occipital strip; POcc, posterior occipital strip. Tracing obtained from the Patient Data Management System associated with the responsive neurostimulation device. (D) Overall trend of long events (>40 sec in length) detected by the responsive neurostimulation device. Bars demonstrate daily event totals. RNS, responsive neurostimulation.

After interdisciplinary team review and with consent, she had RNS device placement on hospital day 74 (NeuroPace RNS, Model RNS‐320; NeuroPace Inc., Mountain View, CA, USA). Two cortical strip electrodes (NeuroPace, Model CL‐325‐10) were placed, one over the right anterior occipital region and a second over the right posterior occipital lobe (Fig. 4A). Stimulation started the next day due to the frequency of seizures that were detected by the device (Fig. 4B), using a lead‐to‐lead configuration with settings of 1.0 mA, 160 µS pulse width, 0.5 µC/Cm^2^ charge density, 100 msec duration, and 200 Hz frequency.

The patient demonstrated gradual improvement and was transferred to a rehabilitation hospital on hospital day 88. A follow‐up PET scan obtained on hospital day 87 showed reduced hypermetabolism as compared to the PET obtained before RNS placement. She was able to engage more meaningfully with her providers and had trace grasp movement in the left hand and trace ability to move her foot. By hospital day 94, she demonstrated a significant decrease in her clinical seizures and in the number of long events detected by the RNS device, defined as events lasting greater than 40 sec (Fig. 4C). Comparing daily long events from hospital days 81–90 (mean: 548.5, standard deviation 67.5) to hospital days 148–157 (mean: 2.4, standard deviation 2.2), there has been a >98% decrease in long events (Wilcoxon Rank‐Sum, *P* < 0.0002). Over this time, her diet was converted to a normal diet, and some of her AEDs were decreased. Genetic and metabolic testing are planned to determine other possible etiologies for her epilepsy.

## Discussion

We present a case in which RNS allowed for control of SRSE that later was consistent with EPC that was refractory to immunomodulatory therapies, ketogenic diet, and rTMS. Due to isolated regions being involved in the invasive recordings, RNS was used to deliver regional therapy, a treatment paradigm that has been described previously.^18^ In addition, RNS will also allow tracking of future treatment responses as she progresses in her recovery.

As reviewed by other authors,^14^, ^19^ deep brain stimulation (DBS), RNS, and vagal nerve stimulation (VNS) have been used as management options for RSE. In few case reports, DBS of the zona incerta,^20^ CM nucleus,^21^, ^22^, ^23^ and the anterior nucleus^24^, ^25^, ^26^ has been used to stop RSE. Cortical stimulation using an adapted DBS device has also been reported in the literature for EPC, though it was not reported whether maximal medical treatment was attempted.^27^ VNS has also been reported in a meta‐analysis to stop RSE and SRSE in 74% of acutely implanted patients, though with limitations of incomplete data and a median 8‐day delay between VNS implantation and SE cessation.^28^

One prior report exists in the literature where RNS was used to treat SRSE in the setting of a focal cortical dysplasia.^29^ However, a change in stimulation settings that resulted in SE cessation occurred concomitantly with the initiation of a ketamine infusion.^19^ Furthermore, while there was no recurrence of SE, seizure frequency returned to pre‐RNS baseline, despite high stimulation therapy. Our case differs in several meaningful ways. First, we demonstrate that RNS can allow for a continued and sustained decrease in detected events, in the setting of a stable medication regimen (Fig. 3C). Importantly, our patient's neurostimulation settings have not been changed. This suggests the possibility that ongoing neuromodulation of the underlying seizure network may be occurring, which has been suggested in long‐term data in RNS patients.^30^, ^31^

There are several limitations to our single case, which required intensive investigation prior to RNS placement. While the patient had evidence of clinical improvement prior to the intracranial study, there were ongoing concerns regarding the progress of her improvement, as well as her persistent clonic movements in the setting of localized periodic EEG patterns. While the patient had presented first with NORSE, this later appeared to be more clinically consistent with a focal motor SE or EPC. It remains unclear whether this later clinical progression made the patient's type of SE more amenable to treatment by neuromodulation.

Due to the nonlesional nature of the case, intracranial recordings were used to understand how the patient's SRSE could be best treated, whether by a regional versus general neuromodulatory approach, though this was debated given her MRI and PET findings. Given CM involvement in thalamocortical networks,^32^, ^33^, ^34^ as well as prior reports of DBS used to treat RSE and the recently demonstrated efficacy of CM‐region RNS in regionalized^35^ and generalized^36^ epilepsy, we placed a thalamic electrode to investigate whether the CM region could be a network target. Ultimately, the CM region did not appear to be involved in the primary organization of the patient's seizure network. In addition, in our case, resective surgery was considered but not pursued due to lack of a clear lesion on imaging, unclear correlate between clonic movements and EEG, and initial semiology involving visual field changes that suggested eloquent cortex could be directly involved in her seizure network.

Finally, while keeping stimulation and detection parameters constant allows dependent therapeutic responses to be tracked accurately, it remains unclear whether these settings could be optimized to speed recovery.^37^ RNS also has technological limitations, such as limited on‐board memory and configuration of two electrodes.

In this case report, we demonstrate the novel, effective use of RNS for treating SRSE in a patient who additionally failed immunomodulatory and rTMS therapies. RNS may be applicable in other patients suffering from SRSE and should be considered in patients with challenging cases of SRSE.

## Conflict of Interest

The MGH Translational Research Center has a clinical research support agreement with Neuralink, Paradromics, and Synchron, for which LRH and SSC provide consultative input.

## Authors' Contributions

J. C. Y. wrote and edited the manuscript and constructed the figures. J. C. Y. and N. M. H. reviewed and described the clinical case details. J. C. Y., N. M. H., and F. A. N. collected the data for the report. V. K. and J. C. Y. worked on 3D reconstruction and figure design. J. C. Y, N. M. H., F. A. N., R. M. R., and S. S. C. conceived the case report. All authors participated in the clinical care and decision making of the patient's care, reviewed the data, and edited the manuscript.
